# A comparison of biofiltration performance based on bacteria and fungi for treating toluene vapors from airflow

**DOI:** 10.1186/s13568-019-0941-z

**Published:** 2020-01-14

**Authors:** Roohollah Ghasemi, Farideh Golbabaei, Sasan Rezaei, Mohammad Reza Pourmand, Ramin Nabizadeh, Mohammad Javad Jafari, Ensieh masoorian

**Affiliations:** 1 Department of Occupational Health Engineering, School of Public Health, Tehran University of Medical Sciences, Tehran, Iran; 2 Department of Medical Mycology & Parasitology, Tehran University of Medical Sciences, Tehran, Iran; 3 Department of Pathobiology, School of Public Health, Tehran University of Medical Sciences, Tehran, Iran; 4 Department of Environmental Health Engineering, Tehran University of Medical Sciences, Tehran, Iran; 5 Safety Promotion and Injury Prevention Research Center, Shahid Beheshti University of Medical Sciences, Tehran, Iran

**Keywords:** Toluene, Elimination capacity, Removal efficiency, Pressure drop, *Pseudomonas putida*, *Pleurotus ostreatus*

## Abstract

With increasing concerns about industrial gas contaminants and the growing demand for durable and sustainable technologies, attentions have been gradually shifted to biological air pollution controls. The ability of *Pseudomonas putida* PTCC 1694 (bacteria) and *Pleurotus ostreatus* IRAN 1781C (fungus) to treat contaminated gas stream with toluene and its biological degradation was compared under similar operating conditions. For this purpose, a biofilter on the laboratory scale was designed and constructed and the tests were carried out in two stages. The first stage, bacterial testing, lasted 20 days and the second stage, fungal testing, lasted 16 days. Inlet loading rates (IL) for bacterial and fungal biofilters were 21.62 ± 6.04 and 26.24 ± 7.35 g/m^3^ h respectively. In general, fungal biofilter showed a higher elimination capacity (EC) than bacterial biofilter (18.1 ± 6.98 vs 13.7 ± 4.7 g/m^3^ h). However, the pressure drop in the fungal biofilter was higher than the bacterial biofilter (1.26 ± 0.3 vs 1 ± 0.3 mm water), which was probably due to the growth of the mycelium. Fungal biofiltration showed a better performance in the removal of toluene from the air stream.

## Introduction

Most of the developing and developed countries are worried about the problems of air pollution (Fazlzadeh Davil et al. 2012; Yunesian et al. 2019). Many of them have approved laws to enforce manufactures to diminish air contaminants release. This is mainly possible by installing air pollution control systems (Fulazzaky et al. 2014).

Biological air pollution controls, which are a suitable alternative to conventional physico-chemical technologies, can convert a variety of compounds (such as VOCs) through the microorganisms activities into harmless elements. Thanks to the high efficiency and environmental friendly features, they are widely used by industries (Hort et al. 2014; Li et al. 2015; Rezaei et al. 2014).

Among the wide diversity of bioreactors (including bio-filters, bio-trickling filters, bio-scrubbers, two-phases partitioning systems, etc.), conventional bio-filters filled with organic materials are suitable for the treatment of the compounds that are hardly solved in water. On the other hand, other bioreactors, in which the liquid phase circulates continuously are recommended to remove compounds with high solubility (Alfonsín et al. 2013; Kennes et al. 2009).

Most biofiltration studies have focused on bacterial activity, and they have shown a great variation and compatibility in the treatment of VOCs (Khammar et al. 2005; Ralebitso-Senior et al. 2012; Schiavon et al. 2016). Although, bacterial biofilters have been introduced as a durable technology for the treatment of gas contaminants, their performance rapidly reduces under conditions of low humidity, low pH, nutrient constraints, and the presence of recalcitrant compounds (Estrada et al. 2013a; Lebrero et al. 2010; Li et al. 2015). On the other hand, some studies based on fungal biofiltration have suggested that this new technology is capable of handling these difficult conditions (Arriaga and Revah 2005; Estrada et al. 2013b; Li et al. 2015; Van Groenestijn et al. 2001).

In general, bacteria are probably more favorable, in optimal conditions, to remove hydrophilic compounds; while fungi can absorb hydrophobic compounds faster than bacterial biofilm (Devinny et al. 1999). In some studies, the bacterium of *Pseudomonas* sp., which has been named in the gaseous BTEX contaminations control processes (Kim and Kim 2005), was also mentioned as one of the bacterial species continuously present in the biofilm of the VOC treatment biofilters (Roy et al. 2003). We only found one study that used a type of fungus called *P. ostreatus* to remove some VOCs (Braun-Lüllemann et al. 1997).

Toluene is a major VOC categorized by the European Commission’s INDEX strategy report and it can be found in indoor air at detectable amounts ranging from a few μg/m^3^ to 358 μg/m^3^ (Baghani et al. 2018; Geiss et al. 2011; Hort et al. 2014; Sarigiannis et al. 2011).

Toluene is widely used in the chemical industry and many operations. Even at low concentrations it can damage the liver and kidneys and cause adverse effects on the central nervous system and the genes (Dehghani et al. 2019; Hazrati et al. 2016a, b; Mohamed et al. 2016; Moro et al. 2012). Toluene is also considered as one of the most difficult VOCs for bio-degradation in gas streams (Hazrati et al. 2015; Wang et al. 2013; Zhao et al. 2014).

In spite of the recent advances in using fungal and bacterial biofiltration, there is a gap in the area of systematic comparative studies on performance evaluation under identical operational conditions. Two separate biofilters for removal of toluene, as a VOC model, were compared in terms of their performances by focusing on two biological processes based on the activity of two types of target microorganisms.

## Materials and methods

### Experimental setup

**Fig. 1:**
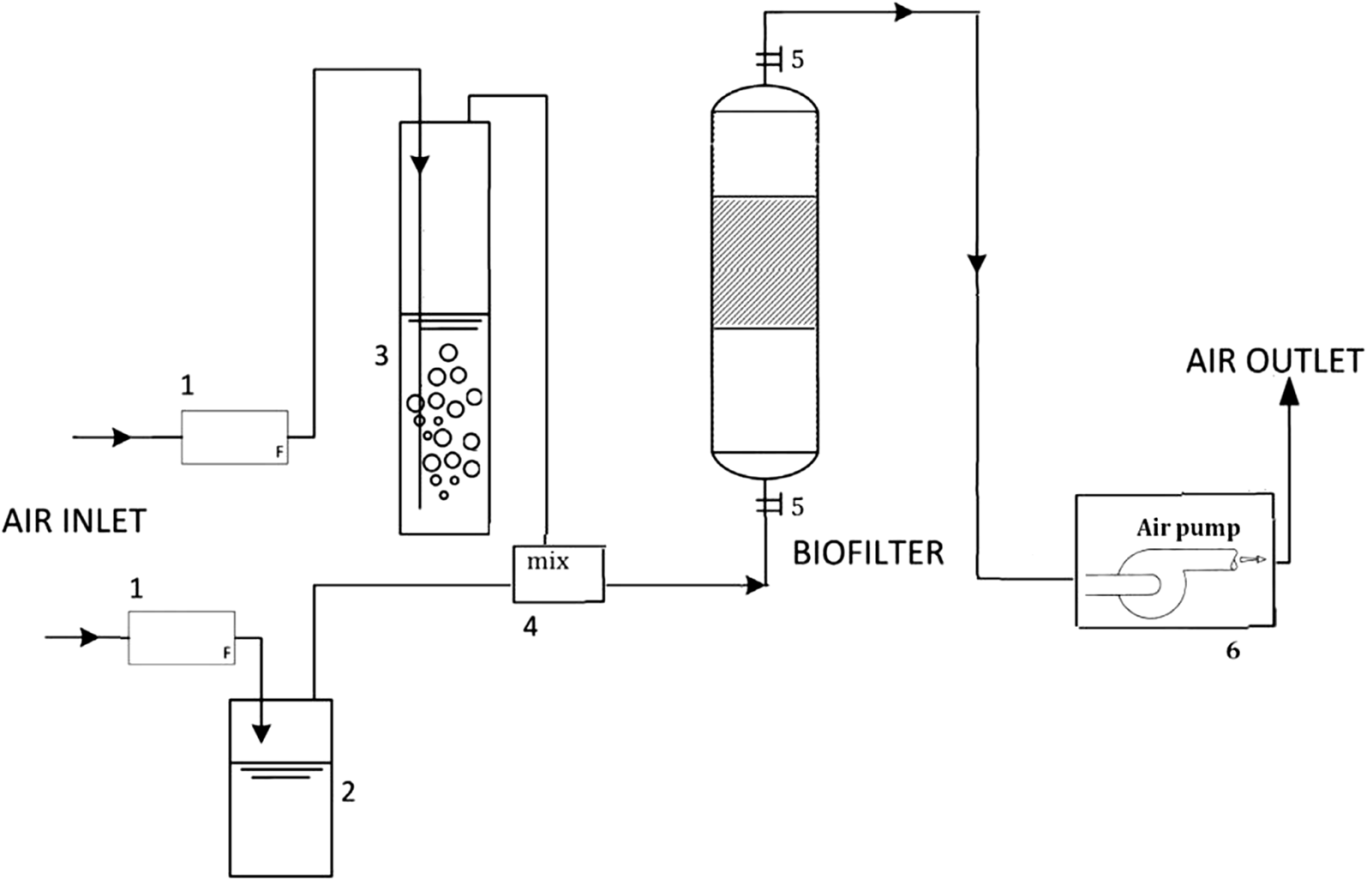
Schematics of the experimental system. 1. Flow meter, 2. impinger containing toluene, 3. humidifier column (bubbler), 4. mixing chamber, 5. gas sampling ports and measuring pressure drop, 6. fan

The experiments were carried out using a laboratory-scale biofilter consisting of a PVC column with an inner diameter of 10.5 cm and a packing height of 8 cm (Fig. 1) with a total packing volume of 0.7 L. Two sampling ports located along the height of the biofilter allowed measurement of the concentration in the inlet and the outlet. Two brazen ports were also located before and after the bed for direct measurement of pressure drop. A fan was used to generate airflow downstream of the biofilter. The flow was divided into main and secondary flows at the beginning of the system, which were both continually monitored by the flowmeter. The larger flow was passed through a water column in order to increase the relative humidity. The humidifier column ID was about 60 mm and height of 60 cm. The stream would enter the mixing chamber, where it is mixed with a smaller stream coming from a 25-mL impinger containing pure toluene (≥ 99%). Therefore, the desired toluene concentrations are achieved by adjusting the micro-valves on the smaller flow path to the impinger. The gas flow rate was adjusted at 2 L/min (with empty bed residence time of 21 s) and the system was operated at 22 ± 2 °C.

### Packing materials and characterization

A mixture of vermi-compost and wood charcoal was used as the packing material with a volume ratio of 2 to 1. The compost particles features were size of 2–4 mm, dry density of 0.302 kg/L, a wet density of 0.628 kg/L, a porosity of 55%, pH between 7 and 8, and moisture content between 44 and 53%. Moreover, the mean size of wood charcoal was 15 ± 2 mm, the void fraction was 50%, the moisture content after 24 h immersion in water was 78%, and the wet and dry densities were 0.8 and 0.44 kg/L respectively.

### Chemicals

Toluene (purity ≥ 99%) used as the target pollutant was purchased from Sigma-Aldrich (Germany). All other chemicals were purchased from Merck (Germany). The mineral salt medium (MSM) used for irrigation in both biofilters was prepared according to Mohamed et al. (2016). The airflow was supplied by a variable-speed model HVDLT-MK2 from SMAKN-CO England.

### Microorganisms and inoculum

#### Bacteria

A pure bacterial culture was used to prepare the inoculum. The strain *P. putida* PTCC 1694 was supplied from the Persian type culture collection (PTCC). It was cultured in nutrient broth and incubated at 30 °C in a rotary shaker (125 rpm, 24 h). The following protocol was developed for the microbial culture in the laboratory: (1) Nutrient broth powder was weighed and dissolved in the 250 mL deionized water in a 1 L bottle and then boiled to achieve a clear solution. (2) It was sterilized in an autoclave for 15 min and at a temperature of 121 °C. (3) After sterilizing and cooling, a mono-colony was inoculated in it with the sterile loop and then it was incubated on a rotary shaker (125 rpm) at 30 °C overnight.

#### Fungi

A pure fungi culture was used to prepare the inoculum. The strain *Pleurotus ostreatus IRAN 1781C* (*Oyster mushroom*) was supplied from the Iranian Research Institute of Plant Protection.

The following protocol was developed for the microbial culture in the laboratory: (1) 7.5 gr nutrient sabouraud dextrose broth powder was dissolved in the 250 mL deionized water in a 1 L bottle and boiled to achieve a clear solution. (2) Then it was sterilized in an autoclave for 15 min and at a temperature of 121 °C. (3) After sterilizing and cooling, a mono-colony was inoculated with the sterile loop and then it was incubated at ambient temperature for 2 days to grow fungus.

#### Experimental conditions

All experiments were performed at 22 ± 2 °C. The experiments were carried out in two different stages, each stage included two phases. The first stage lasted for 20 days. In order to ensure that only the microorganism’s activity, not other mechanisms such as adsorption, affects the pollutants, the sterilized bed (30 min, 121 °C) was filled in the biofilter (BF) with the mentioned ratio to allow adsorption tests. The sterilized BF was operated for 10 days and exposed to a wide range of inlet loading ratio (LR) of toluene. The average LR during these 10 days was 21.9 ± 8.11 g/m^3^ h. At the end of the tenth day, the bed was inoculated with 100cc of a pre-prepared *Pseudomonas putida*-rich bacteria solution. After bacterial inoculation, the operation lasted for another 10 days. The average loading rate for these 10 days was 21.27 ± 4.53 g/m^3^ h.

The second stage was similar to the first one and it was aimed to investigate the ability of the fungi to remove toluene from the air stream. However, this stage lasted for 16 days and like the first stage, the same sterile bed was used in the first 6 days of operation. The average LR during these first 6 days was 24.54 ± 10.6 g/m^3^ h. At the end of the 6th day, the bed was inoculated with 100 cc of a pre-prepared *Pleurotus ostreatus*-rich fungi solution. The operation continued for up to 10 days. The average inlet load for these 10 days was 27.26 ± 5 g/m^3^ h. It is worth noting that the experiments were carried out in a full-factorial design; consisting of 207 runs for bacteria and 158 for fungi. All tests were performed at least 5 times a day for at least 3 consecutive days. The other factors such as temperature, flow rate, Empty Bed Residence Time (EBRT), column size and diameter, type and amount of packed material, irrigation and nutrient solution, media moisture content and so on were the same during these two stages of testing.

To control bed humidity, 30 mL of mineral nutrient solution consisting of macro elements (g L^−1^): K_2_HPO_4_ 1; KH_2_PO_4_ 1; KNO_3_ 1; NaCl 1; MgSO_4_ 0.2; and micro element (mg L^−1^) ZnCl_2_ 0.07; MnCl_2_·2H_2_O 100; CaCl_2_·2H_2_O 26; FeCl_3_·4H_2_O 1.3; H_3_BO_3_ 0.06; NiCl_2_·6H_2_O 0.025; CoCl_2_·2H_2_O 0.12; CuCl_2_·2H_2_O 0.015; Na_2_MoO_4_·2H_2_O 0.025 were supplied once per 3 days. The pressure drop was measured by U-water-filled manometers connected to the inlet and outlet of the biofilter. In addition, 100 μL gaseous samples were drawn by Gastight Hamilton syringes from the sampling ports and then analyzed with GC.

#### Gas chromatography analysis

To determine the RE of the system, toluene vapor concentration was measured upstream and downstream at BF and the analyses were carried out continuously by GC-FID (model CP-3800 gas chromatograph and FID detector, Varian Technologies Japan Inc., Japan) and a capillary column (25 m × 0.25 mm × 0.25 µm). The injector, detector, and oven temperatures were set at 200, 240 and 130 °C respectively. As the carrier gas, N_2_ was used (1.8 mL/min) and 100 µL of the air sample was injected into the injection port with a split ratio of 5. The standard concentrations of toluene were made in TEDLAR sample bags and injected to GC to sketch the calibration curve (R^2^ = 0.999).

## Results

### Toluene removal efficiency in bacterial BF

**Fig. 2:**
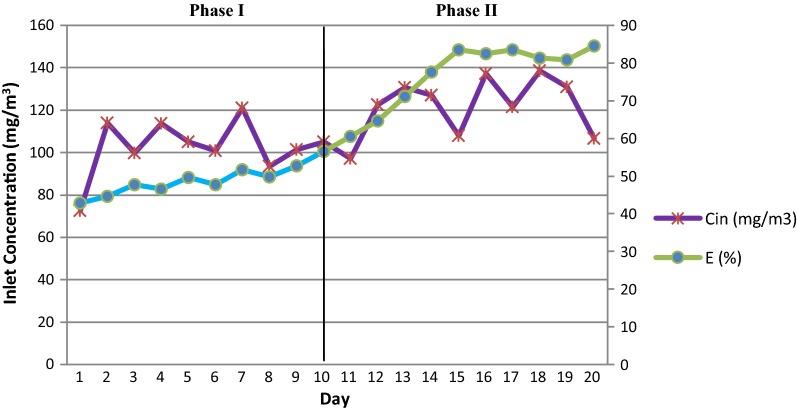
Bacterial BF efficiency changes versus inlet toluene concentration during 20 days of operation. Phase I and Phase II are sterilized and inoculated bed respectively

Removal efficiency (RE) variations in bacterial BF versus toluene inlet concentration are shown in Fig. 2. During the 20 days of operation, the removal efficiency was gradually increased, which was slower and inconsiderable in the first phase (first 10 days without inoculation). During this time, the minimum and maximum inlet loading rates were 12.85 and 38.3 g/m^3^ h respectively. The LRs were unstable during the 10 consecutive days. Inlet load on the first and tenth days were 19.51 and 18.87 g/m^3^ h respectively, and the corresponding removal efficiencies were 44.95 and 57.10% respectively.

As noted, inoculation took place at the end of the 10th day. It should be noted that inoculation was carried out by toluene-adapted bacteria. The performance trend increased more quickly after the inoculation. This trend was continued for another 5 days, and on the 15th day, the performance graph was almost flat and stable. During the second 10 days (the second phase), the inlet contaminations were also fluctuant. Inlet loads on the 11th and 20th days were 15.11 and 18.8 g/m^3^ h respectively. In addition, the corresponding removal efficiencies on the 11th and 20th days were 62.74 and 82.2% respectively. The results showed that the removal efficiency during the second phase reached a maximum of 82.11%, which was observed on day 17.

The data about average inlet contaminant concentration (C_in_), Inlet loading rate (LR), removal efficiency (RE), elimination capacity (EC) during 20 days of the first stage is given in Table 1.

**Table 1:** Data for first stage (Phase I, Phase II)—bacterial biofiltration

Phase	Day	C_in_ (mg/m^3^)	LR (g/m^3^ h)	EC (g/m^3^ h)	RE (%)
1 (Blank)	1–10	126.8 ± 46.8	21.9 ± 8.11	11.2 ± 4.5	50.6 ± 4.3
2 (Case)	11–20	122.8 ± 19.2	21.27 ± 3.3	16.23 ± 3.37	75.8 ± 7

### Data for first stage (Phase I, Phase II)—bacterial biofiltration

#### Toluene removal efficiency in fungal BF

**Fig. 3:**
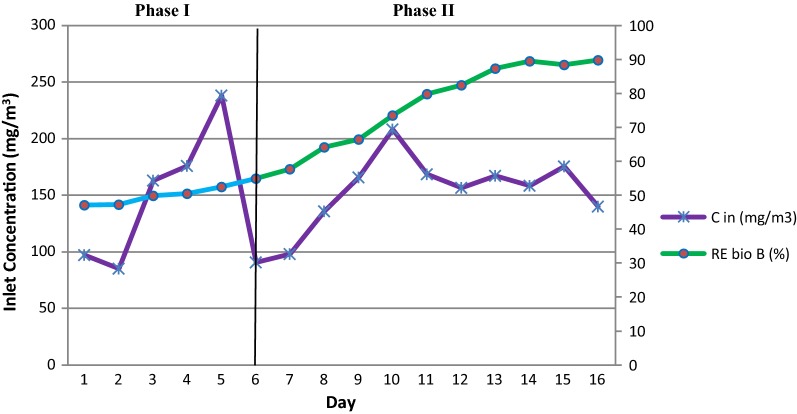
Fungal BF efficiency changes versus inlet toluene concentration during 16 days of operation. Phase I and Phase II are sterilized and inoculated bed respectively

Removal efficiency (RE) changes in fungal BF vs. the inlet concentration during the entire operating time are shown in Fig. 3. This stage lasted 16 days and the results demonstrated the same trends as with the bacterial BF. The trend starts with a slow increase and then the efficiency increases with a higher slope after the fungi inoculation. During the phase I of the second stage, the minimum and maximum inlet toluene loading rates were 14.73 to 30.43 g/m^3^h respectively. The LRs on the first and 6th days were 16.84 and 15.74 g/m^3^ h respectively and the corresponding removal efficiencies on these 2 days were 47.14 and 54.9% respectively.

Inoculation was carried out at the end of the 6th day by toluene-adapted fungi. Clearly, the efficiency trend increased after the inoculation as it reached 79.8% on the 11th day. Afterwards, the growing trend slowed down and the efficiency graph can be considered stable and uniform. The inlet loads on the 7th and 16th days were 17 and 24.26 g/m^3^ h respectively, and the corresponding removal efficiencies were 57.7 and 89.82% respectively. The highest removal efficiency during phase II was observed in the second stage in the last day.

The data about the average inlet contaminant concentration (C_in_), Inlet loading rate (LR), removal efficiency (RE), and elimination capacity (EC) during 16 days of the second stage is given in Table 2.

**Table 2:** Data for second stage (Phase I, Phase II)—fungal biofiltration

Phase	Day	C_in_ (mg/m^3^)	LR (g/m^3^ h)	EC (g/m^3^ h)	RE (%)
1 (Blank)	1–6	141.7 ± 61.15	24.54 ± 10.6	12.44 ± 5.67	50.35 ± 3
2 (Case)	7–16	157.4 ± 28.76	27.26 ± 5	21.47 ± 5.42	77.94 ± 11.8

### Second stage (Phase I, Phase II)—fungal biofiltration

#### Bed pressure drop in both experiments

**Fig. 4:**
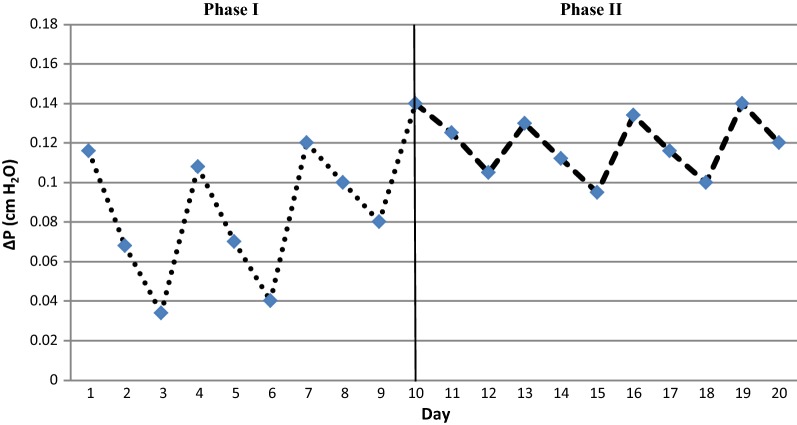
Bed pressure drop as a function of time in the bacterial BF. Phase I and Phase II are sterilized and inoculated bed respectively

**Fig. 5:**
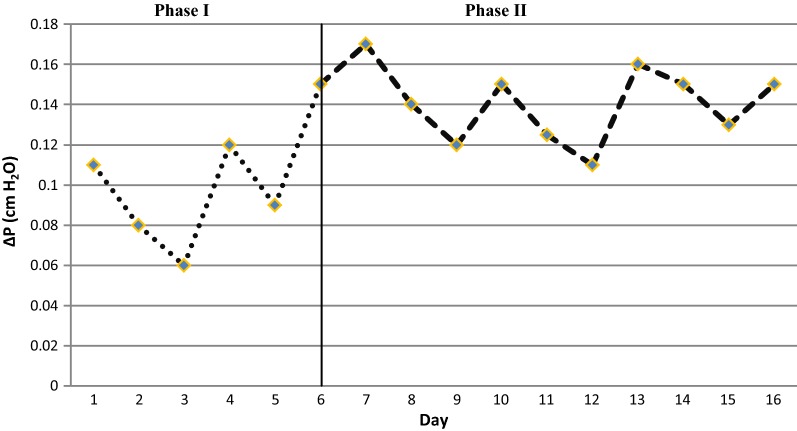
Bed pressure drop as a function of time in the fungal BF. Phase I and Phase II are sterilized and inoculated bed respectively

Pressure drop variations of the bacterial and fungal BFs are shown in Figs. 4 and 5 respectively. The mean pressure drops during phases I and II of the first stage of experiment were 0.87 ± 0.35 and 1.2 ± 0.15 mm water respectively. The mean pressure drops during phases I and II of the second stage were 1 ± 0.3 and 1.4 ± 0.2 mm water respectively. The pressure drop curves in the first and second phases of both stages are specified with dots and dashes respectively. The mean pressure drop in both stages of the experiment showed an increasing trend.

## Discussion

### Toluene removal efficiency in both BFs

Toluene was used as a model pollutant because it is a hydrophobic contaminant and hardly biodegradable; therefore, it can be a representative compound for VOCs (Wang et al. 2013; Zhao et al. 2014). The results of toluene removal in bacterial and fungal BFs (Figs. 2 and 3) demonstrated that sterilized bed (first phase) did not have a high efficiency in toluene removal and at best, it only removed 57.1% of toluene in the first stage and 54.9% in the second stage. During the first phase the REs were only due to packing materials adsorption. This result is consistent with the findings of the Klapková et al. (2006) study. These efficiency values were maintained during the first phases since the BFs were aerated at night (during the experiments) for 12 h with the minimum flow and pollutant concentration. This can regenerate the removal of additional toluene (Mohamed et al. 2016). Ondarts et al. (2012) showed that the compost was a poor adsorbent (3 × 10^− 3^ μg/g) when they were used for toluene treatment at a concentration of 81 μg/m^3^ (Ondarts et al. 2012).

After inoculation (the beginning of the second phase), the bacterial BF was able to remove about 82.11% after only 5 days, and the fungi BF was able to remove about 82.5% of organic load after 6 days. Therefore, the average removal efficiency in the bacterial and fungi BFs was maintained at 81% and 87.5% respectively. That is, steady state conditions were achieved. Rahul et al. (2013) demonstrated that a steady state condition was presumed when the variations in the pollutant removal efficiency were within 5% for 3 sequential days.

As shown in Figs. 2 and 3, after inoculation, the efficiency increased with a slight gradient, which could be an indicative of the adaptation of the microorganisms to the contaminants and the existing conditions. However, the adaptation period was not long in this study since the pre-adapted microorganisms were used (Amin et al. 2014). The adaptation time in other studies ranges from a few days to a few weeks and a month (Estrada et al. 2013a; Klapková et al. 2006; Novak et al. 2008).

Comparing the performance of the bacterial and fungal BFs (Figs. 2 and 3, Tables 1 and 2), it turns out that the fungus was more effective in eliminating toluene contaminant. In addition, Li et al. (2015) observed a high percentage of removal (99%) of ethyl-mercaptan, styrene, alphapinene, and sulfur compound mixture in a fungal BF inoculated with the species *P. ostreatus*.

Studies have also focused on the structure of the fungi and the pollutant capturing mechanism in their mycelia and confirmed that it can directly absorb hydrophobic compounds (Krailas et al. 2000). The reason for this is that there is no water layer between fungi biofilm and gas phase; therefore, hydrophobic compounds are removed faster comparing with bacterial biofilm (Fulazzaky et al. 2014; Pedersen et al. 1997).

Estrada et al. (2013a, b) compared fungal and bacterial biofiltration for the treatment of a VOC mixture including toluene (Estrada et al. 2013a). They concluded that bacterial biofiltration showed higher removal efficiencies and mineralization ratios than its fungal peer (27.7 ± 8.9 vs 40.2 ± 5.4 g/m^3^ h), However, different species were used in their study.

#### Bed pressure drop in both experiments

Figures 4 and 5 illustrate a slight increase in the pressure drop curves during the two stages of the operation, which indicates the subsidence and compression of the bed especially in the first phase of the experiments (Chen et al. 2013; Estrada et al. 2013b; Klapková et al. 2006). In addition, the formation and gradual growth of the biomass and mycelium cause partial obstruction of the pores of the media, and it is a factor in the more pressure drop during the second phases in two stages (Dorado et al. 2012; Padhi and Gokhale 2014; Schiavon et al. 2016).

Compared to the bacterial, the fungal BF exhibited a higher average pressure drop; which is also confirmed by some studies. As (Van Groenestijn and Liu 2002) suggested, the high pressure drop in a fungal BF was due to the occupation of void spaces by mycelium, which is considered as one of the main defects in fungal biofiltration (Van Groenestijn and Liu 2002). However, the maximum pressure drop after 60 days of the experiment was of 912 Pa/m bed, which is acceptable for industrial scale (Estrada et al. 2013b; Van Groenestijn and Liu 2002).

The presence of the mites was confirmed by microscopic observations of biofilm samples in a bacterial BF, but not in a fungal BF. This explains the lower pressure in the bacterial BF (Woertz et al. 2002). Estrada et al. (2013) stated that bacterial biofiltration showed final values 60% less than those of fungal biofiltration, which is economically considerable (Estrada et al. 2013a).

It is also clear that the curves in both stages of the experiments were of saw shapes, which represent the irrigation periods that were sprayed on the bed every 3 days. Studies have shown that the accumulation of water and moisture in the pores causes blockage and clogging of the bed and thus creates resistance against the airflow (Amin et al. 2014; Dorado et al. 2010; Kawase et al. 2014).

To the best of our knowledge, this study may be the first to compare fungal and bacterial biofilters based on *Pseudomonas putida* and *Polarotus straus*, in terms of toluene removal capacity in VOC model and pressure drop. In general, fungal biofilter showed a higher elimination capacity and mineralization rates than that of the bacterial biofilter. However, the pressure drop in the fungal biofilter was higher than that in the bacterial biofilter. Given the higher efficiency and the higher pressure drop in fungal biofilters, there is a need for studies on the cost-effectiveness and energy consumption of these two processes. In this study, only toluene was used as a pollutant and future works can focus on the treatment of a mixture of VOCs.

## Data Availability

All data are presented in figures and tables within this article. Any material used in this study will be available for research purposes upon request.
